# IGL-1 preservation solution in kidney and pancreas transplantation: A systematic review

**DOI:** 10.1371/journal.pone.0231019

**Published:** 2020-04-02

**Authors:** Melanie Habran, Julie De Beule, Ina Jochmans

**Affiliations:** 1 Department of Microbiology, Immunology, and Transplantation, Lab of Abdominal Transplantation, Transplantation Research Group, KU Leuven, Leuven, Belgium; 2 Abdominal Transplantation Surgery, University Hospitals Leuven, Leuven, Belgium; Imperial College Healthcare NHS Trust, UNITED KINGDOM

## Abstract

We aimed to systematically review published data on the effectiveness of Institut Georges Lopez-1 (IGL-1) as a preservation solution for kidney and pancreas grafts. A systematic literature search of PubMed, Embase, Web of Science, and the Cochrane Library databases was performed. Human studies evaluating the effects of IGL-1 preservation solution in kidney and/or pancreas transplantation were included. Outcome data on kidney and pancreas graft function were extracted. Of 1513 unique articles identified via the search strategy, four articles could be included in the systematic review. Of these, two retrospective studies reported on the outcome of IGL-1 compared to University of Wisconsin (UW) solution in kidney transplantation. These show kidneys preserved in IGL-1 had improved early function (2 weeks post-transplant) compared to UW. Follow-up was limited to 1 year and showed similar graft and patient survival rates when reported. Two case series described acceptable early outcomes (up to 1 month) of simultaneous kidney pancreas transplantation after storage in IGL-1. As only four clinical papers were identified, we widened our search to include four eligible large animal studies. Three compared IGL-1 with UW in pig kidney transplant models with inconclusive or mildly positive results. One pig pancreas transplant study suggested better early outcome with IGL-1 compared to UW. Too few published data are available to allow any firm conclusions to be drawn on the effectiveness of IGL-1 as a preservation solution of kidney and pancreas grafts. The limited available data show satisfactory early outcomes though no medium to long-term outcomes have been described. Further well-designed clinical studies are needed.

## Introduction

Static cold storage of donor organs remains the main method to preserve solid organs. It allows affordable organ preservation and unsupervised shipment of organs. During static cold storage, donor organs are first flushed with a cold preservation solution. They are subsequently immersed in this preservation solution and stored on melting ice at 0°C to 4°C.

As the organ cools down, metabolism is slowed down [1] and the detrimental effects of oxygen and nutrient deprivation are diminished. However, the harmful effects of oxygen deprivation, although reduced, are not completely halted. Oxygen deprivation leads to the depletion of cellular adenosine triphosphate causing impairment of ionic pumps, cell swelling, and eventually cell death. Furthermore, the rapid cooling in itself has harmful effects. As the phospholipid layer undergoes changes induced by hypothermia, membranes become stiffer and lose their selective permeability leading to loss of cellular function and integrity. [2]

It is clear that organs need to be protected during the cold ischemic phase of static cold storage. Therefore, preservation solutions, especially designed to combat the detrimental effects of both ischemia and hypothermia, were developed. Belzer and Southard noted that in order for a solution to be effective, it must consist of a composition that (1) minimizes cell swelling induced by hypothermia, (2) prevents intracellular acidosis, (3) prevents the expansion of interstitial space during flush-out, (4) prevents injury from reactive oxygen species (ROS), and (5) provides substrates for regenerating high-energy phosphate compounds during reperfusion. [3] Based upon these principles, several other cold storage solutions were developed and introduced in the clinical setting. [4] Each preservation solution has its own unique mix of components that counteract interstitial and cellular edema, cellular acidosis and production of reactive oxygen species. [5] To the current day, the University of Wisconsin (UW) solution remains the gold standard solution against which all others are measured.

Today, abdominal organs are most often preserved by either UW or Histidine-Tryptophan-Ketoglutarate (HTK). [6] IGL-1, or Institut Georges Lopez-1 solution, is a relatively new player on the market that is increasingly used in Europe. The large, flexible, water-soluble polymer polyethylene glycol (PEG) serves as impermeant in IGL-1 and is capable of creating high osmotic pressures while it is unlikely to interact with biological chemicals. Because of its further similarities with UW, IGL-1 is sometimes called the UW-PEG solution. Indeed, apart from an extracellular Na/K concentration and PEG instead of hydroxyethyl starch, IGL-1 is very similar to UW (Table 1), though it has several theoretical advantages. [7] Hydroxyethyl starch, UW’s impermeant, is associated with aggregation of red blood cells and this, in combination with its high viscosity, could lead to poor flush out of donor organs during procurement. [8–10] The viscosity of IGL-1 is lower than that of UW though still higher than the non-viscous HTK, so that lower volumes of IGL-1 are sufficient to ensure its efficacy as a flush-out solution during donor procedures. [8, 11] PEG also has been shown to reduce influx of CD4+ and CD8+ inflammatory T-cells after perfusion. [12, 13] PEG might even reduce ROS-induced damage as reduced lipid peroxidation was observed in isolated hepatocytes and in an isolated kidney perfusion model. [14, 15] Additionally, PEG has also been shown to protect mitochondrial integrity. [12] IGL-1, has an extracellular-like composition with low potassium concentrations which has been shown to reduce vasospasm, related to potassium-induced smooth muscle cell depolarization. [16]

**Table 1:** Composition of the preservation solutions UW, HTK and IGL-1. [7].

	UW	HTK	IGL-1
**Sodium**	30 mM	15 mM	120 mM
**Potassium**	120 mM	9 mM	25 mM
**Calcium**	-	0.0015 mM	0.5 mM
**Chloride**	20 mM	32 mM	-
**Impermeant**	Lactobionate Raffinose Hydroxyethyl starch (50g/L)	Mannitol	Lactobionate Raffinose Polyethylene glycol 35kDa (1g/L)
**Buffer**	Phosphate	Histidine	Phosphate
**ROS scavenger**	Gluthatione Allopurinol	Tryptophan	Gluthatione Allopurinol
**Nutrients**	Adenosine	Ketoglutarate	Adenosine

IGL-1, Institut Georges Lopez solution; HTK, histidine-tryptophan-ketoglutarate; ROS, reactive oxygen species; UW, University of Wisconsin solution; kDa, kiloDalton

With this systematic review we aimed to summarize the published clinical data on the effectiveness of IGL-1 as a preservation solution for kidney and vascular pancreas grafts.

### Methods

This systematic review was performed according to the Preferred Reporting Items for Systematic Reviews and Meta-Analyses (PRISMA) guidelines. Inclusion and exclusion criteria as well as the search strategy were registered in the International Prospective Register of Systematic Reviews (PROSPERO) prior to the literature search (registration number: CRD42019128259). The possibility of a meta-analysis had been foreseen in case enough eligible and qualitative articles were available, though this was not the case and therefor a meta-analysis was not carried out.

### Search strategy

PubMed, Embase, Web of Science and the Cochrane Library were searched for articles discussing the use of IGL-1 to preserve kidney and/or vascular pancreas grafts in clinical transplantation. The search strategy was set up in collaboration with an experienced librarian (S1 and S2 Tables).

As IGL-1 does not exist as a MesH term or Emtree term, we used only one concept–“IGL”–to prevent too much narrowing of the search results. If the concepts “kidney” and “pancreas” had been added to the search strategy, relevant articles might have been missed because of a too specific search strategy. All relevant synonyms of IGL-1 were used and combined by the Boolean operator “OR”. Inclusion and exclusion criteria were prepared in advance (S3 Table). Clinical reports on post-transplant outcomes of kidney and/or vascular pancreas grafts cold-stored in IGL-1 were considered. Articles were excluded when they discussed in vitro work or animal studies (both in vivo and ex vivo); were written in a language other than English, Dutch or French; had no full text available; or fit the following study types: review articles, letters, editorials, or conference abstracts. Outcomes of interest were delayed graft function (DGF), primary non function, graft survival, graft function (evolution of serum creatinine, daily urine output, creatinine clearance).

Two independent researchers performed the search. The results of all database searches were uploaded in Endnote (Version X7.8 (MH) or X9 (JDB), Clearview Analytics, Philadephia, PA, USA) and pooled together in one library to be used by each independent researcher. The resulting articles were screened for duplicates using the “Find Duplicates” tool in EndNote (S4 Table). After removal of duplicates, the remaining articles were screened on title and abstract based on inclusion and exclusion criteria. All of this was done by two independent researchers (MH, JDB). Afterwards, eligibility screening was also performed independently by the same two researchers. Articles evaluating the effect of IGL-1 preservation on outcome after kidney or pancreas transplantation were considered as eligible. At least, they should focus on any of the outcome parameters of interest: DGF, rejection, graft or patient survival after kidney or pancreas transplantation. Ideally, they would compare the use of IGL-1 to another preservation solution. As literature was so limited, descriptive studies were also accepted as eligible. Non-eligible articles were further excluded. In case of any dissimilarity, the differences were discussed between the two researchers. A third experienced researcher (IJ) was available in case of any discrepancies, though all discrepancies could be solved between the two independent researchers.

Of the remaining eligible articles included in this study, a data extraction table was made containing the following items: authors, title, journal, year of publication, kidney and/or pancreas study, the type of study (prospective/retrospective, (matched) case-control/case series/longitudinal studies), type of cold storage solution (CSS), the number of included patients, type of donor, selected donor and recipient characteristics (age, cold ischemia time), occurrence of DGF, occurrence of rejection, non-insulin dependent status after transplantation (in case of pancreas transplantation), follow-up time, and graft and patient survival.

### Expansion of the search selection criteria

Because only very few articles were found to fit the eligibility criteria, additional steps were taken to ensure maximal inclusion of relevant clinical articles and avoid missing articles because of a publication bias. These consisted of (1) “snowballing” by searching the reference lists of the included articles for studies fitting the inclusion criteria; (2) screening of records excluded based on language criteria; (3) screening conference abstracts; (4) running the search separately on clinicaltrials.gov and the WHO’s International Clinical Trials Registry Platform to identify ongoing trials/unpublished results. Any records that fit the inclusion criteria were added to the initial search. We further broadened our inclusion criteria to accept studies evaluating the effectiveness of IGL-1 in large animals as these might provide additional clinically relevant data. In the group of kidney transplantation the possibility of a meta-analysis was explored. Although cold ischemia times and the comparison between IGL-1 and UW were identical, the animal models and outcome measures were too different to perform an adequate quantitative synthesis of the results.

### Quality and risk of bias assessment

The Jadad scoring tool was to be used to assess the methodological quality of clinical trials. For retrospective case-control series and case series, where the Jadad score is not an ideal quality assessment tool, the respective National Institutes of Health (NIH) scoring tools were used. [17] Risks of bias were assessed following the advice of the Cochrane Collaboration by individually assessing each study using a simple judgment of low risk, high risk or unclear risk for the following risk of bias: selection, performance, detection, attrition, and reporting bias. Risks of bias are therefore assessed at study level and not on outcome level. We also report the level of evidence based on the evidence pyramid for each study included. [18] For animal studies, quality and risk of bias assessment was performed by using the Systematic Review Centre for Laboratory animal Experimentation (SYRCLE’s) risk of bias tool. [19]

## Results

**Fig 1:**
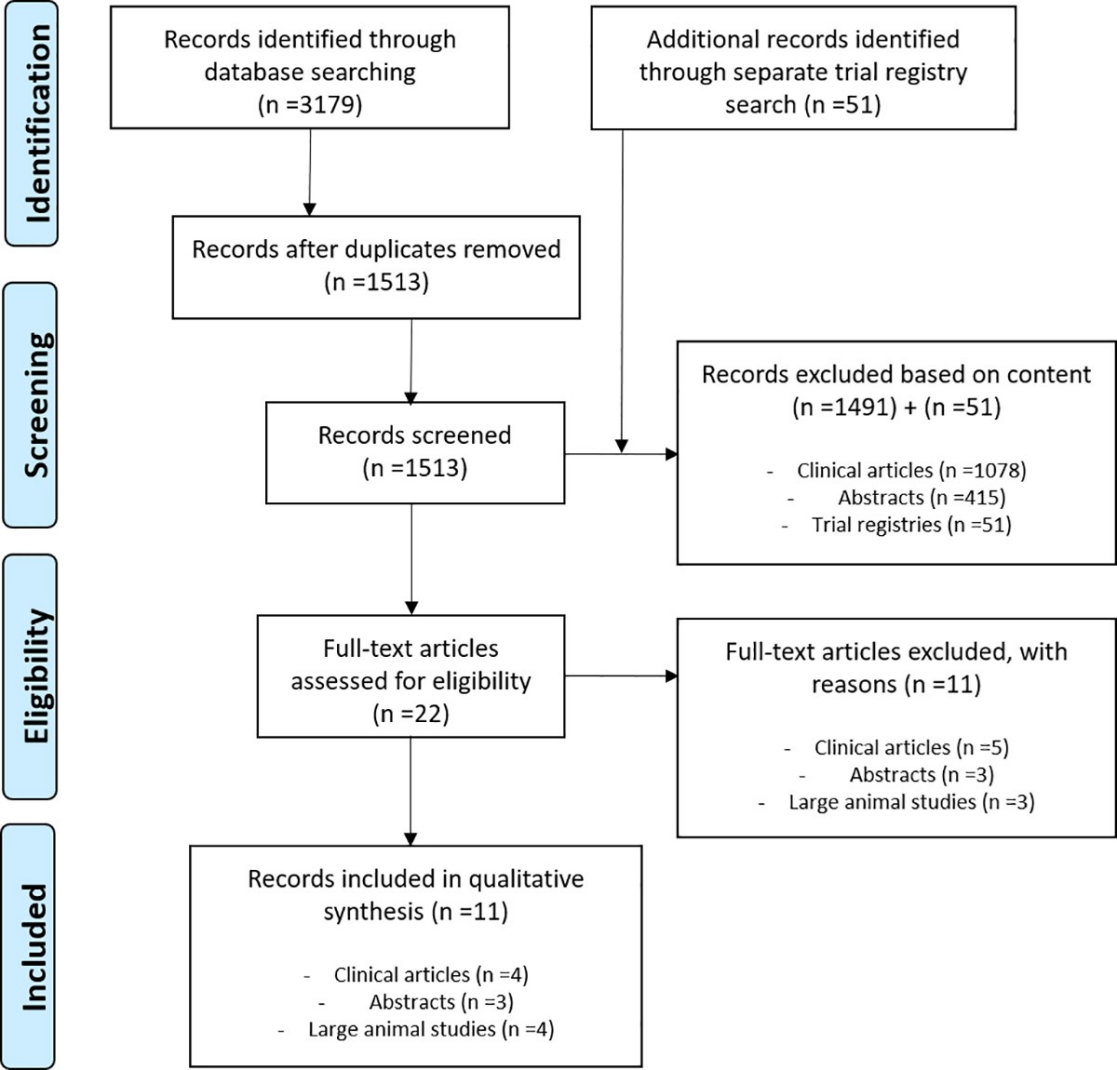
Flowchart.

The search was performed on March 18^th^, 2019. In the search 3179 articles were identified (PubMed: 894, Embase: 1279, Web of Science: 985, and Cochrane Library: 21 articles of which 20 are trials) (Fig 1). After removal of duplicates, 1513 unique articles remained which were screened on title and abstract. Based on content 1491 records (1078 articles and 415 abstracts) were excluded.

The full text of the 22 remaining records was screened on the basis of which another eleven records were excluded based on content. Three of the excluded records were conference abstracts and the other eight were articles of which three animal studies. One of these three excluded animal studies was a duplicate in another language of the included record. [20, 21]

Eleven records were eventually included in this systematic review of which four clinical retrospective studies. Two articles reported on clinical trials using IGL-1 in kidney transplantation [22, 23]; two discussed case series of the use of IGL-1 in simultaneous kidney pancreas transplantation. [24, 25] Broadening the search resulted in three abstracts and four animal studies included in this systematic review. Additional snowballing and searching of registry databases (on January 15^th^, 2020) separately could not identify any additional records or ongoing or unpublished trials.

### Quality assessment and risk of bias

Both articles concerning the use of IGL-1 in kidney transplantation were case-control studies (level 3 studies) scoring 3 out of 12 points on the NIH scoring tool for case-control studies (S5 Table). [22, 23]

The quality of the case series (level 4 studies) on simultaneous kidney pancreas transplantation, assessed with the NIH tool for case series, was good (S6 Table). [24, 25] In both case series the study objective and the study population were clearly defined. The intervention and the outcome measures were also clearly described. All studies included in this systematic review showed an overall high risk of bias (S7 and S8 Tables).

The abstracts identified in the extended search mostly reported the same cohorts of patients as in the included articles except for the study of Darius et al. [26–28] Information available in abstracts is limited and this is reflected in the quality of the work. Included large animal studies did not report about sequence generation, allocation concealment and selective outcome reporting (S9 and S10 Tables). Although this complicates assessment of selection, detection and reporting bias, risk of performance bias could be assessed as low in all articles reporting about outcome after kidney transplantation.

### The use of IGL-1 in kidney transplantations

Both articles reporting on the use of IGL-1 as a preservation solution in kidney transplantation concern retrospective studies. [22, 23] In these, kidney preservation with IGL-1 is compared to UW. As recipient outcome data are from overlapping eras, both studies most likely include, at least in part, the same patient population. In these studies, reported baseline characteristics are comparable (Table 2). Both studies reported renal function outcome based on the following outcome measures: DGF, creatinine clearance during the first 2 postoperative weeks and then at 1, 3, 6 and 12 months, daily urine output and evolution of creatinine. As an additional outcome measure, acute rejection episodes were also reported in the latest article from 2009.

**Table 2:** Overview of outcome measures for the articles reporting on kidney transplantation.

Authors	Study type	Evidence level	Era	Patient N°	Donor type	Donor age (y)	Recipient age (y)	Cold ischemia time (h)	DGF	Rejection	Graft survival	Patient survival
Badet et al [22]	Retrospective non-matched case-control study	3	27/06/2003-30/06/2004	IGL-1: 37 UW: 33	Deceased donor	IGL-1: 40±14 UW: 42±14	IGL-1: 44±11 UW: 48±11	IGL-1: 16±4 UW: 17h±6	IGL-1: 6% (2/37) UW: 14% (6/33)	NR	NR	NR
Codas et al [23]	Retrospective non-matched case-control study	3	06/2003-12/2004	IGL-1: 121 UW: 102	Deceased donor	IGL-1: 39 (16–70) UW: 44 (16–72)	IGL-1: 49 (19–71) UW: 50 (18–73)	IGL-1: 17 (9–34) UW: 16 (9–44)	IGL-1: 13% (16/121) UW: 13% (13/102)	IGL-1: 12% (15/121) UW: 13% (13/102)	IGL-1: 98% UW: 99%	IGL-1: 98% UW: 100%
Darius et al [28]	Abstract	**-**	01/01/2014-30/08/2017	IGL-1: 33 UW: 62	Living donor	NR	NR	NR	IGL-1: 0% UW: 0%	IGL-1: 6% (2/33) UW: 10% (6/62)	IGL-1: 98% UW: 100%	IGL-1: 100% UW: 100%

Outcome measures are reported as mean±SD or median (min-max). DGF, delayed graft function; IGL-1, Institut Georges Lopez preservation solution; NR, not reported; UW, University of Wisconsin preservation solution

Badet et al report on 70 kidney transplant recipients of which 37 were preserved by IGL-1 that were followed for one year. [22] In this cohort study, post-transplant serum creatinine values–as measured between 2 and 14 days post-transplant–were significantly lower in the IGL-1 group compared to the UW group. Serum creatinine concentrations were also significantly lower in the first month post-op in the IGL-1 group, but no other time points showed a significant difference. DGF rates were also reported to be lower after preservation with IGL-1 compared to UW (respectively 6% and 14%). Graft and patient survival were not reported in this study.

In 2009, Codas et al reported on 232 kidney transplant recipients of which 121 were preserved with IGL-1 that were followed for one year. [23] Here it is reported that median serum creatinine values (from 6 to 14 days post-op) were significantly lower in the IGL-1 group compared to UW. Also, the creatinine values declined significantly faster in the IGL-1 group from 4 to 15 days post-transplant. Creatinine clearance was significantly lower in the first 15 postoperative days in the IGL-1 group compared to UW. Kidney function at other time points was similar in both groups. The incidence of DGF was the same in both IGL-1 and UW (13% vs. 13%, respectively). Those recipients experiencing DGF after receiving a kidney that was preserved by IGL-1 only needed one dialysis session in 53% of cases, while this was the case in 46% of recipients that had received a kidney preserved with UW. The incidence of acute rejection episodes was similar in both groups (12% in the IGL-1 group vs. 13% in the UW group). At one-year follow-up, patient survival rate was 98% in the IGL-1 group and 100% in the UW group. Graft survival was 98% in the IGL-1 group vs. 99% in the UW group.

Of the two eligible abstracts identified through the extended search, one reported on the patient population described in the paper by Codas et al. [26] Darius et al showed data comparing IGL-1 (n = 33) with UW (n = 62) preservation of kidneys from living donor procedures (Table 2). [28] No primary non-function or DGF occurred in any of the living donor kidney transplants. Incidence of acute rejection was 6.2% in the IGL-1 group and 9.7% in the UW group. Graft survival rates were 98% for the IGL-1 group and 100% for the UW group. Reason of graft loss was not reported in the abstract. Patient survival rates were 100% in both groups.

Three relevant large animal studies could be identified after broadening the search (Table 3). [21, 29, 30] Badet et al reported on a pig auto transplant model where kidneys were subjected to 24 hours of cold ischemia. [21] Early function was evaluated by measuring daily creatinine, creatinine clearance, fractional sodium excretion and urea levels. On day 6 and 7 significant lower creatinine values were reported in the IGL-1 group when compared to UW. A significant decrease in Major Histocompatibility Complex (MHC) class II molecules, number of apoptotic cells and percentage of surface area labelled by alpha-smooth muscle actin (SMA) was reported for the IGL-1 group in comparison to the UW group. In two studies on a low mismatch allograft pig model with 24 hours of cold preservation of the kidney and no immunosuppression, no major differences between IGL-1 and UW were found when looking at 1 and 3 months post-transplant kidney function, animal survival, and tissue changes (tubulo-interstital fibrosis, influx of inflammatory cells). [29, 30]

**Table 3:** Overview of outcome measures for the animal articles reporting on kidney transplantation.

Authors	Animal model	N° animals per group	CIT (h)	Longest FU	Endpoints
**Badet et al [21]**	Pig autotransplant model	IGL-1 (n = 6) K-UW (n = 6) Sham (n = 4)	24	7 days	○Kidney function (creatinine (clearance), FRNa, urea) ○IHC: MHC class 2 expression (mAb)–interstitial fibrosis (alpha-SMA)Histology (injury scoring) and cellular apoptosis (TUNEL)
**Thuiller et al [29]**	Low mismatch allograft pig model	IGL-1 (n = 6) UW (n = 6) SCOT (n = 6) Sham (n = 6)	24	3 months	○Kidney function (serum creatinine, urine production) ○Histology and cellular apoptosis (TUNEL) ○Interstitial fibrosis (Picro Sirius staining) ○IHC: MHC class 2 expression (mAb)–VEGF- HIF alfa, MCP-1, beta 2 microglobine, …
Thuillier et al [30]	Low mismatch allograft pig model	IGL-1 (n = 18) UW (n = 18) SCOT (n = 18)	24	3 months	○Kidney function (serum creatinine, proteinuria) ○Interstitial fibrosis (Picro Sirius) + Inflammatory cells (CD3+, monocytes, macrophages) ○long-term hypoxic injury (HIF-alfa, VEGF, EPO) ○Epithelial-to mesenchymal transition development

alpha-SMA, alpha-smooth muscle actin; CD3, cluster of differentiation; CIT, cold ischemia time; EPO, erythropoietin; FRNa, fractional sodium excretion; FU, follow-up; HIF, hypoxia inducible factor; IGL-1, Institut Georges Lopez preservation solution; IHC, immunohistochemistry; mAb, monoclonal antibody; MHC, major histocompatibility complex; MCP-1, monocyte chemoattractant protein-1; SCOT, solution de conservation des organs et des tissus; TUNEL, terminal dUTP-transferase-mediated nick end labelling; UW, University of Wisconsin preservation solution; VEGF, vascular endothelial growth factor

### The use of IGL-1 in simultaneous kidney pancreas transplantation

Chedid et al described 5 consecutive cases of simultaneous kidney and pancreas transplantation using IGL-1 as a cold storing solution (Table 4). [24] All pancreata acquired normal function and insulin independency immediately after reperfusion. Three out of five patients developed DGF of the kidney graft. One patient presented with acute cellular rejection, limited to the pancreas, which was successfully treated with 5 doses of intravenous thymoglobulin. One patient died on day 10 due to cardiogenic shock with normal pancreas and kidney function, the other four patients were alive with functioning grafts at last follow-up though actual follow-up time was not reported.

**Table 4:** Overview of outcome measures for the case series reporting on simultaneous pancreas and kidney transplantation.

Authors	Study type & level of evidence	Time era	Patient N°	Donor type	Donor age (y)	Recipient age (y)	Cold ischemia time (h)	DGF	Immediate function pancreas graft	Independent of insulin	Rejection	Patient survival	Graft survival
Chedid et al [24]	Case report (level 4)	02–2015–10–2015	5	Not reported	18, 29, 35, 18 and 38	29, 26, 35, 23 and 50	Kidney: 14, 11, 13, 7.5 and 6.5 Pancreas: 17, 13, 14, 9.2 and 9.7	60% (3/5)	Yes	All	None	80% (4/5)	100%
Igreja et al [25]	Case series (level 4)	01/2012–9/2017	46	Not reported	26 ± 8.5	36 ± 7	13h ± 3	Not reported	Yes	All	None	96% (44/46)	93.6%

Outcome measures are reported as mean ± standard deviation. DGF, delayed graft function

Igreja et al reported 47 cases of, in all but one, simultaneous kidney and pancreas transplantation (Table 4). [25] This study includes 46 patients, one patient needed retransplantation after loss of the pancreas graft 2 years after the first transplantation and received a pancreas preserved by IGL-1. In all patients, normalization of pancreatic function occurred early after reperfusion, all kidneys functioned immediately, and all patients maintained a non-insulin dependent status after transplantation. Pancreatic graft loss followed by patient death occurred in two cases (one due to pancreatic thrombosis and one due to sepsis). Another patient presented with graft loss due to pancreatic thrombosis, who was later retransplanted. One patient died with a functioning graft on the 34^th^ day after transplantation due to sepsis from an infected hematoma. At one-month follow-up, pancreas graft survival was 44 out of 47 (94%). One patient presented with post-transplantation pancreatitis characterized by elevated amylase values and changes in ultrasonography, which resolved following unspecified treatment. Patient survival after one-month follow-up time was 44 out of 46 patients (96%). Igreja et al did not report on outcomes concerning the kidney graft, whereas Chedid et al reported 3 cases on 5 who developed renal DGF (definition of DGF was not given). Our extended search identified one abstract by Chedid et al, reporting on the same four cases with less detail. [27]

A pilot study published by Garcia-Gil et al reports on a model of vascular pancreas allotransplantation in pigs where pancreata were transplanted after 16 hours of cold ischemia, stored in either IGL-1 (n = 8) or UW (n = 8). [8] Outcomes were: graft function (defined as normoglycemia for at least 5 days) and peak amylase / lipase levels. All grafts of the IGL-1 group functioned (8/8, 100%) whereas only six did in the UW group (6/8, 75%) as a results of vascular thrombosis. Peak amylase and lipase levels after IGL-1 preservation were about half of the levels seen in the UW group, although no statistical significance was found. Eventually all grafts failed because of acute cellular rejection, which was confirmed by Banff grading.

## Discussion

This is the first systematic review to summarize the published clinical data on the effectiveness of IGL-1 as a preservation solution for kidney and pancreas grafts. IGL-1 is a relatively new preservation solution used for static cold storage of abdominal organs. This systematic review shows that there is limited data available in the literature reporting on the immediate outcomes of kidneys and pancreata preserved with IGL-1 and no clinical studies looking at long-term outcomes. The available clinical studies are either case series with a follow up time of one month or retrospective analyses that likely report on considerable patient overlap with a follow up time of one year. Inherent to the design of the included studies (level 3 or 4) risk of bias was assessed as high in all of them. Furthermore, quality of the case-controlled level 3 evidence was found to be poor in contrast to the case series that scored rather well. Taking all of the above into account, the scarce clinical data published need to be interpreted with caution. The few large animal studies available add little additional information as groups are small, comparisons are difficult, and results are inconclusive.

The reported immediate outcomes after clinical transplantation of kidneys preserved by IGL-1 are good and even reported to be significantly better than kidneys preserved by UW. This is in line with reported preclinical data in a kidney autotransplant model in pigs where kidneys preserved in IGL-1 solution showed improved early function and a significant reduction of MHC class II expression, cellular apoptosis and interstitial fibrosis compared to UW. [21] However, these results were not confirmed in pig allotransplant models. [29, 30]

There was no difference in graft and patient survival one year after transplantation in the reported clinical retrospective studies. There are currently no data published on the long-term outcomes of kidneys preserved by IGL-1 in human, either as case series or in comparison to other preservation solutions.

The clinical case series reporting on IGL-1 used in pancreas transplantation suggest that IGL-1 is a safe and effective solution. [24, 25] In both articles no pancreas dysfunction was noted and all patients became independent of insulin after transplantation. Pancreas preservation with IGL-1 of up to 17 hours was reported. [24] These findings seem to match the scarce preclinical data studying IGL-1 in pancreas transplantation. When IGL-1 was compared to UW in a model of pig pancreas transplantation, it offered the same degree of safety and effectiveness as UW. [8] There are no reports on the long-term outcomes of pancreas transplantation after preservation with IGL-1.

As IGL-1 is an abdominal preservation solution, and liver, kidneys, pancreas and intestines are flushed with the same solution during organ procurement, data about efficacy of IGL-1 on outcomes after liver transplantation are also important. A recent systematic review of Szilagyi et al concluded that UW, HTK and IGL-1 solutions are associated with nearly equivalent outcomes. [31] Subgroup analyses could not identify any differences between IGL-1 and UW or HTK for primary non function and overall graft survival after one year. Some caution is warranted as only 5 studies were available for inclusion in this systematic review and therefore no firm conclusion can be drawn.

As with all systematic reviews, it is possible that relevant articles were not found although this is unlikely given the fact that a broad search strategy was set up in collaboration with an experienced librarian. Furthermore, as clinical work proved to be scarce, we expanded our search as widely as possible to include published abstracts, reports in other languages as well as large animal studies. Risk of bias was assessed as high for all clinical studies, stressing that available data should be interpreted with caution. Articles reporting on large-animal studies had a low risk on performance and detection bias. However, many details with regard to sequence generation, allocation concealment and selective outcome reporting were not reported, making it difficult to assess risk of selection and reporting bias adequately.

In conclusion, the limited published clinical data seems to suggest that IGL-1 is a safe and promising preservation solution for the static cold storage of kidney and pancreas grafts. However, because there are no large data series available, it is currently unclear whether outcomes after transplantation of kidneys and pancreata preserved with IGL-1 are equivalent to those obtained with UW or other commonly used abdominal preservation solutions. Additional well-designed studies and, ideally randomized controlled trials, are needed to demonstrate equivalence or superiority of one solution over the other.

## Supporting information

S1 Checklist PRISMA 2009 checklist. (DOC)

S1 Table Summary of the PICO process. (DOCX)

S2 Table Search strategy for the data sources used: PubMed, Embase, Web of Science and the Cochrane Library. (DOCX)

S3 Table Inclusion and exclusion criteria. (DOCX)

S4 Table Fields selected in the EndNote Find Duplicates tool with number of found duplicates per step. (DOCX)

S5 Table Quality assessment using the NIH scoring tool for case-control studies. (DOCX)

S6 Table Quality assessment of the case series using the NIH scoring tool for case series. (DOCX)

S7 Table Risk of bias assessment of all included studies. (DOCX)

S8 Table Risk of bias assessment in detail. (DOCX)

S9 Table Risk of bias assessment animal studies. (DOCX)

S10 Table Risk of bias assessment in detail animal studies. (DOCX)
