# Supplementary material for: IGL-1 preservation solution in kidney and pancreas transplantation: A systematic review
Source: PLoS One. 2020 Apr 2;15(4):e0231019. doi: 10.1371/journal.pone.0231019 (PMC7117741; doi:10.1371/journal.pone.0231019)
Supplement: S1 Table — (DOCX) [file pone.0231019.s002.docx]

**S1 Table. Summary of the PICO process.**

| Population | Kidney and/ or vascular pancreas transplant recipients |
| --- | --- |
| Intervention | IGL-1 preservation solution |
| Comparison | None, UW, or HTK preservation solution |
| Outcome(s) | delayed graft function, primary non function, graft survival, graft function (, evolution of serum creatinine, daily urine output, creatinine clearance) |
