# Supplementary material for: IGL-1 preservation solution in kidney and pancreas transplantation: A systematic review
Source: PLoS One. 2020 Apr 2;15(4):e0231019. doi: 10.1371/journal.pone.0231019 (PMC7117741; doi:10.1371/journal.pone.0231019)
Supplement: S2 Table — (DOCX) [file pone.0231019.s003.docx]

**S2 Table. Search strategy for the data sources used: PubMed, Embase, Web of Science and the Cochrane Library.**

| Database | Search strategy |
| --- | --- |
| PubMed | "IGL-1 solution"[Supplementary Concept] OR “IGL-1”[tiab] OR “Institut Georges Lopez”[tiab] OR (Institut[tiab] AND George[tiab] AND Lopez[tiab]) OR “IGL”[tiab] OR IGL1[tiab] |
| Embase | ‘IGL-1’:ti,ab,kw OR ‘Institut Georges Lopez’:ti,ab,kw OR (Institut:ti,ab,kw AND George:ti,ab,kw AND Lopez:ti,ab,kw) OR ‘IGL’:ti,ab,kw OR IGL1:ti,ab,kw |
| Web of Science | “IGL-1” OR “Institut Georges Lopez” OR (Institut AND George AND Lopez) OR “IGL” OR IGL1 |
| Cochrane | (“IGL-1” OR “Institut Georges Lopez” OR (Institut AND George AND Lopez) OR “IGL” OR IGL1):ti,ab,kw |
| Trial registries  *(search expansion)* | ClinicalTrials.gov: “IGL-1” OR “Institut Georges Lopez” OR (Institut AND George AND Lopez) OR “IGL” OR IGL1  WHO’s International Clinical Trials Registry Platform: (Institut AND George AND Lopez OR “Institut Georges Lopez” OR “IGL” OR IGL1) |
