# Supplementary material for: IGL-1 preservation solution in kidney and pancreas transplantation: A systematic review
Source: PLoS One. 2020 Apr 2;15(4):e0231019. doi: 10.1371/journal.pone.0231019 (PMC7117741; doi:10.1371/journal.pone.0231019)
Supplement: S3 Table — (DOCX) [file pone.0231019.s004.docx]

**S3 Table. Inclusion and exclusion criteria.**

| Inclusion criteria | - Clinical (human) studies (in vivo or ex vivo) reporting on post-transplant outcomes of kidney and/or vascular pancreas grafts cold stored in IGL-1 - Studies in English, Dutch or French - Articles with full text available - Research articles which are no review articles, letters, editorials, abstracts |
| --- | --- |
| Exclusion criteria | - In vitro studies - Preclinical/animal studies (in vivo and ex vivo) - Language other than English, Dutch or French - Articles with no full text available - Review articles - Letters, Editorials, Abstracts |
