# Supplementary material for: IGL-1 preservation solution in kidney and pancreas transplantation: A systematic review
Source: PLoS One. 2020 Apr 2;15(4):e0231019. doi: 10.1371/journal.pone.0231019 (PMC7117741; doi:10.1371/journal.pone.0231019)
Supplement: S4 Table — (DOCX) [file pone.0231019.s005.docx]

**S4 Table. Fields selected in the EndNote Find Duplicates tool with number of found duplicates per step.**

| Step | Selected Fields | Duplicates Found |
| --- | --- | --- |
| 1 | Author, Year, Title, Journal (Secondary Title) | 441 |
| 2 | Author, Year, Title, Pages | 181 |
| 3 | Title, Journal (Secondary Title), Pages | 222 |
| 4 | Title, Volume, Issue, Pages | 46 |
| 5 | Author, Year, Journal (Secondary Title), Pages | 101 |
| 6 | Author, Year, Volume, Issue, Pages | 18 |
| 7 | Author, Title | 312 |
| 8 | Year, Volume, Issue, Pages | 70 |
| 9 | Title | 234 |
| 10 | Author, Year | 69 |
