# Supplementary material for: IGL-1 preservation solution in kidney and pancreas transplantation: A systematic review
Source: PLoS One. 2020 Apr 2;15(4):e0231019. doi: 10.1371/journal.pone.0231019 (PMC7117741; doi:10.1371/journal.pone.0231019)
Supplement: S5 Table — (DOCX) [file pone.0231019.s006.docx]

**S5 Table. Quality assessment using the NIH scoring tool for case-control studies.[1]**

|  | Badet et al. [2] | Codas et al. [3] |
| --- | --- | --- |
| Clear study question / objectives | Y | Y |
| Study population clearly described | N | N |
| Sample size justification | N | N |
| Cases and controls from same population | N | N |
| Consistent use definitions/criteria to identify cases | N | N |
| Cases clearly differentiated from controls | Y | Y |
| If less than 100% eligible cases/controls selected, were randomly selected from those eligible | N | N |
| Use of concurrent controls | N | N |
| Able to confirm exposure/risk prior to the development of condition | N | N |
| Measures of exposure/risk clearly defined, implemented consistently | Y | Y |
| Assessors blinded | N | N |
| Key potential confounding variables measured/matching | N | N |
| Total points | 3 | 3 |
| Items are scored as yes (Y), no (N), cannot determine (CD), not applicable (NA), not reported (NR) | | |
