# Supplementary material for: IGL-1 preservation solution in kidney and pancreas transplantation: A systematic review
Source: PLoS One. 2020 Apr 2;15(4):e0231019. doi: 10.1371/journal.pone.0231019 (PMC7117741; doi:10.1371/journal.pone.0231019)
Supplement: S6 Table — (DOCX) [file pone.0231019.s007.docx]

**S6 Table. Quality assessment of the case series using the NIH scoring tool for case series.[12]**

|  | Chedid et al. [14] | Igreja et al. [15] |
| --- | --- | --- |
| Clear study question / objectives (Y/N) | Y | Y |
| Study population clearly described (Y/N) | Y | Y |
| Consecutive cases (Y/N) | Y | Y |
| Comparable subjects (Y/N) | Y | Y |
| Clearly described intervention (Y/N) | Y | Y |
| Clearly defined outcome measure (Y/N) | Y | Y |
| Adequate length of follow-up (Y/N) | N | Y |
| Well described statistical methods (Y/N) | N | N |
| Well described results (Y/N- | Y | Y |
| Total points | 7 | 8 |
| Items are scored as yes (Y), no (N), cannot determine (CD), not applicable (NA), not reported (NR) | | |
