# Supplementary material for: IGL-1 preservation solution in kidney and pancreas transplantation: A systematic review
Source: PLoS One. 2020 Apr 2;15(4):e0231019. doi: 10.1371/journal.pone.0231019 (PMC7117741; doi:10.1371/journal.pone.0231019)
Supplement: S7 Table — (DOCX) [file pone.0231019.s008.docx]

**S7 Table. Risk of bias assessment of all included studies.**

|  | Badet et al 2005 | Codas et al 2009 | Chedid et al 2016 | Igreja et al 2018 |  |
| --- | --- | --- | --- | --- | --- |
|  | - | - | - | - | **Selection bias** |
|  | - | - | - | - | **Performance bias** |
|  | - | - | - | - | **Detection bias** |
|  | - | - | - | - | **Attrition bias** |
|  | ? | ? | ? | ? | **Reporting bias** |
|  |  |  |  |  |  |
| Legend | - | high risk | | |  |
|  | + | low risk | | |  |
|  | ? | unclear risk | | |  |
