# Supplementary material for: IGL-1 preservation solution in kidney and pancreas transplantation: A systematic review
Source: PLoS One. 2020 Apr 2;15(4):e0231019. doi: 10.1371/journal.pone.0231019 (PMC7117741; doi:10.1371/journal.pone.0231019)
Supplement: S8 Table — (DOCX) [file pone.0231019.s009.docx]

**S8 Table. Risk of bias assessment in detail.**

|  | **KIDNEY** | | | **PANCREAS** | |
| --- | --- | --- | --- | --- | --- |
|  | **Badet et al 2005** | | **Codas et al 2009** | **Chedid et al 2016** | **Igreja et al 2018** |
| **Selection bias** | | population specified clearly, although selection bias always at risk in retrospective study | probably 1/4 is the same cohort as study of Badet et al | population specified clearly, although selection bias always at risk in retrospective study | population specified clearly, although selection bias always at risk in retrospective study |
| **Performance bias** | higher risk as it is no RCT | | higher risk as it is no RCT | few cases, selected afterwards | no control group |
| **Detection bias** | only 1y FU for long-term outcomes | | only 1y FU for long-term outcomes | FU period not specified | only 1month FU  for long-term outcomes |
| **Attrition bias** | higher risk in retrospective analyses  (missing data for example) | | matching not clear | higher risk in retrospective analyses  (missing data for example) | higher risk in retrospective analyses  (missing data for example) |
| **Reporting bias** | no negative effects reported | | no negative effects reported | no control group to compare | no control group to compare |
