# Supplementary material for: IGL-1 preservation solution in kidney and pancreas transplantation: A systematic review
Source: PLoS One. 2020 Apr 2;15(4):e0231019. doi: 10.1371/journal.pone.0231019 (PMC7117741; doi:10.1371/journal.pone.0231019)
Supplement: S9 Table — (DOCX) [file pone.0231019.s010.docx]

**S9 Table. Risk of bias assessment animal studies.**

|  |  | Badet et al 2005 | Thuillier et al 2011 Transplantation | Thuillier et al 2011 Br J Surg | Garcia-Gil et al 2014 |
| --- | --- | --- | --- | --- | --- |
| **Selection bias** | Sequence generation | ? | ? | ? | ? |
|  | Baseline characteristics | + | + | + | - |
|  | Allocation concealment | ? | ? | ? | ? |
| **Performance bias** | Random housing | + | + | + | ? |
|  | Blinding | ? | + | + | ? |
| **Detection bias** | Random outcome assessment | ? | ? | - | + |
|  | Blinding | ? | + | + | ? |
| **Attrition bias** | Incomplete outcome data | - | - | ? | + |
| **Reporting bias** | Selective outcome reporting | ? | ? | ? | ? |
| **Other bias** | other issues | + | + | + | + |
|  |  |  |  |  |  |
| Legend |  |  |  |  |  |
| - | high risk |  |  |  |  |
| + | low risk |  |  |  |  |
| ? | unclear risk |  |  |  |  |
