# Supplementary material for: IGL-1 preservation solution in kidney and pancreas transplantation: A systematic review
Source: PLoS One. 2020 Apr 2;15(4):e0231019. doi: 10.1371/journal.pone.0231019 (PMC7117741; doi:10.1371/journal.pone.0231019)
Supplement: S10 Table — (DOCX) [file pone.0231019.s011.docx]

**S10 Table. Risk of bias assessment in detail animal studies.**

|  |  | KIDNEY | | | PANCREAS |
| --- | --- | --- | --- | --- | --- |
|  |  | Badet et al 5/2005 Transpl Int | Thuillier et al 3/2011 Transplantation | Thuillier et al 3/2011 Br J Surg | Garcia-Gil et al 5/2014 Transplantation |
| **Selection bias** | Sequence  generation | random component described without further information | not clearly described reference to procedure: there randomization | not clearly described | random component described without further information |
|  | Baseline  characteristics | creatinine values similar in all groups (also sham) | creatinine values similar in all groups (also sham) | same time points for shams, no baseline for each pig | no baseline values, no sham |
|  | Allocation  concealment | randomization was not clear as well as concealment | as randomization was not clear concealment could not be evaluated | as randomization was not clear concealment could not be evaluated | as randomization was not clear concealment could not be evaluated |
| **Performance bias** | Random housing | pigs housed according national guidelines +  effect on outcome unlikely | pigs housed according national guidelines +  clear description | all-in metabolic cage | not specified |
|  | Blinding | not specified in the text | surgical teams were  blinded to protocols | surgical teams were  blinded to protocols | not described |
| **Detection bias** | Random outcome assessment | not specified in the text | not specified in text | 6 animals sacrificed per time point randomization not clear | not random, but at glucose levels >150mg/dl for 2 consecutive days |
|  | Blinding | not specified in the text | all sections examined  under blinded conditions | all sections  under blinded conditions for | not described |
| **Attrition bias** | Incomplete outcome data | one pig died because of acute renal failure just left out of the analysis but this could have an effect on outcome creatinine | animals lost from  surgical complications excluded not clear how they handled this in the analyses | not clearly described if there were any losses | difference between analysis with and without thrombosis failures |
| **Reporting bias** | Selective  outcome  reporting | not clear if there was a protocol | not clear if there was a protocol | not clear if  there was a protocol | ethical committee protocol |
| **Other bias** | other | no other | no other | no other | no other |
